# Less time spent walking and depressive symptoms are associated with more self-perceived disability in primary care patients: a cross-sectional study from Uganda

**DOI:** 10.11604/pamj.2022.41.173.30453

**Published:** 2022-03-03

**Authors:** James Mugisha, Peter Kayiira Byansi, Philip Ward, Davy Vancampfort

**Affiliations:** 1 Department of Sociology and Social Administration, Kyambogo University, Kampala, Uganda,; 2 Africa Social Development and Health Initiatives, Kampala, Uganda,; 3 Faculty of Health Sciences, Uganda Martyrs University, Kampala, Uganda; 4 School of Psychiatry, University of New South Wales, Sydney, NSW, Australia,; 5 Schizophrenia Research Unit, Ingham Institute of Applied Medical Research, Liverpool, NSW, Australia,; 6 KU Leuven Department of Rehabilitation Sciences, Leuven, Belgium,; 7 University Psychiatric Centre KU Leuven, Leuven-Kortenberg, Belgium

**Keywords:** Depression, disability, exercise, holistic health

## Abstract

**Introduction:**

evidence on associations between self-perceived disability and physical activity levels in primary care patients (PCP) in low-income countries is absent. We investigated whether self-perceived disability is associated with physical activity levels while taking into account relevant demographic, social, mental and health parameters and other lifestyle factors in PCP in Uganda.

**Methods:**

in this cross-sectional study, patients from two primary care centers in a farming community in central Uganda completed the World Health Organization Disability Assessment Schedule 2 (WHODAS 2), Simple Physical Activity Questionnaire (SIMPAQ), Patient Health Questionnaire-9 (PHQ-9), Alcohol Use Disorders Identification Test, and Multidimensional Scale for Perceived Social Support. Somatic co-morbidity and multimorbidity were self-reported or retrieved from medical files. A backward linear regression was performed in order to explain the variance in WHODAS 2 total scores.

**Results:**

in 130 PCP [median (interquartile range) age=47.0 (22.0); 73.1% (n=95) female], older age, less time spent walking (SIMPAQ) and more severe depressive symptoms (PHQ-9) were independent significant predictors of more self-perceived disability (WHODAS 2). The final model explained 44.2% of the variance in WDODAS 2 scores.

**Conclusion:**

our study demonstrates that self-perceived disability in PCP living in low-resourced settings is associated with older age, physical inactivity and depressive symptoms. Future lifestyle studies in primary care settings should consider targeting both physical and mental health outcomes in order to reduce self-perceived disability in PCP, in particular in older patients.

## Introduction

Non-communicable diseases (NCDs) are responsible for almost 70% of the world´s deaths per year [1]. About three quarters of these NCD-related deaths occur in low- and middle-income countries (LMICs) [1]. In particular Africa is projected to have the greatest increase in NCDs burden by 2025 [1], and NCDs will bypass infectious diseases as the most important cause of premature mortality in this part of the world [2]. Reasons for this transition are among others a trend towards an increasingly elderly population and lifestyle changes including unhealthy diets and physical inactivity [3]. Despite this increasing burden, the primary care quality in low-income countries to manage NCDs is challenged by inadequate facility infrastructure and equipment, and limited human resources [4]. Low-cost and easy implementable interventions are needed in primary care settings, not only to reduce the NCD burden, but also the associated self-perceived disability [5-8]. Self-perceived disability can be defined as experiencing difficulties in carrying out daily activities, challenges with social inclusion and uncertainty or worry about future health [9]. It may be consistent, episodic or variable over time [9]. Episodes of self-perceived disability can be exacerbated by intrinsic factors (for example the presence of mental health symptoms) and extrinsic factors (such as lack of social support) [9].

Although in high-income countries the treatment in primary care settings has shifted in recent years towards a more integrated and holistic approach that includes diminishing self-experienced disability via lifestyle interventions such as physical activity, evidence on associations between self-perceived disability and physical activity levels in primary care patients (PCP) in low-income countries is currently still absent. Exploring this association in low-income countries is important given differing levels of knowledge regarding the benefits of physical activity [10] and environmental factors (e.g., safety, climate) [11] compared to high-income countries. If a significant association between self-perceived disability and physical activity levels would be found, this would, according to the behavioural epidemiological framework [12], provide evidence to the support development and evaluation of physical activity interventions within primary care settings to reduce self-perceived disability in PCP.

NCDs are also in Uganda an increasing public health burden. They are estimated to contribute to one third of all deaths, while cardiovascular diseases alone contribute to one tenth of these premature deaths [13]. Although the majority of the Ugandan population is complying with the international physical activity recommendations of the World Health Organization [14,15], the majority of vulnerable people attending primary care centers such as those suffering from a chronic condition including cardiovascular diseases [15-17] and mental illness [18] are not. In particular people with two or more chronic conditions (i.e. multimorbidity), which is increasingly being seen in Uganda [19] are at a high risk for inactivity [20]. Since no study to date has examined the association of self-perceived disability with physical activity levels in PCP in a low-income country, the aim of the current study was to fill this gap in the literature and to assess whether self-perceived disability is associated with physical activity levels in PCP in Uganda. In exploring the association between self-perceived disability and physical activity levels in PCP in Uganda, we will take into account relevant demographic (age, gender), social (levels of social support) [21], mental health (i.e. symptoms of depression) [21,22], physical health (i.e. presence of a chronic condition, multimorbidity) [21,22], malnutrition [23,24] and other lifestyle (i.e. harmful drinking, smoking) variables that are known to be associated with self-perceived disability and physical activity levels. We hypothesize that self-perceived disability will be negatively associated with physical activity levels, even after taking into account relevant demographic, social, mental health, physical health, and lifestyle variables in Ugandan PCP.

## Methods

**Study design**: we conducted a cross-sectional study exploring the association between self-perceived disability and physical activity levels in Ugandan PCP.

**Study setting:** this study was executed in two primary care centers in the Mpigi health district in Central-Uganda.

**Study population:** the population in Mpigi health district in Central-Uganda is predominantly semi-literate (80%), involved in subsistence farming (59%) and small-scale retail businesses [25].

**Study sampling:** all people who were attending one of the involved primary care centers during the two-weeks study period were invited to participate by a trained clinical officer. Eligible were all primary care patients aged 18 or older, irrespective of the reason for attending the primary care setting.

### Study variables

**Demographical and clinical variables:** patients were asked whether they had a paid job (yes versus no). Age, gender, and the presence of chronic somatic comorbidities (yes versus no and which condition) were self-reported and, where possible, confirmed via the medical files. Multimorbidity was considered being present if two or more chronic somatic comorbidities were reported. For calculating the body mass index (BMI), weight was measured in light clothing to the nearest 0.1 kg using a SECA beam balance scale, and height to the nearest 0.1 cm using a wall-mounted stadiometer. Malnutrition was identified as having a BMI lower than 18.5 [26]. Participants were asked whether they smoked or not, and if so, how many cigarettes were smoked per day on average.

World Health Organization Disability Assessment Schedule 2 (WHODAS 2). The Luganda version of the WHODAS 2 [27] was used to assess the levels of self-perceived disability. It gathers information across six domains, i.e., cognition, mobility, self-care, getting along, life activities, and participation in society, asking about difficulties in these domains during the 30 days preceding the interview. Disability perception is measured by responses on a 5-point scale from 1 (no difficulty) to 5 (extreme difficulty or cannot do). The WHODAS 2.0 algorithm was used to compute an overall score total score which ranges from 0 to 100 with a higher score indicating greater self-perceived disability. The WHODAS 2 has a good internal consistency reliability, test-retest reliability, and construct validity in people with chronic conditions in Sub-Saharan Africa [28] and has been used in primary care patients [29].

**Simple Physical Activity Questionnaire (SIMPAQ):** the Luganda version of the SIMPAQ [30] was used. The SIMPAQ is a 5-item clinical tool to assess physical activity levels among populations at high risk for physical inactivity. It uses an interview format to estimate time spent in bed (min/day), time spent sedentary during waking hours (min/day), time spent napping (min/day), time spent walking (min/day), time spent time spent in structured exercise (min/day), and time spent in incidental or non-structured physical activity (min/day) during the past week. The sum of the hours recorded in the SIMPAQ items should add to approximately 24-hours, providing interviewers with an opportunity to clarify with participants if significant under or over-reporting has occurred (e.g., total of < 18 hours or > 30 hours accounted for). In this study we focus on the time spent walking, time spent time spent in structured exercise, and time spent in incidental or non-structured physical activity. Previous research in people with mental and physical health problems in Uganda demonstrated the questionnaire is reliable [31] while the validity has been demonstrated in a 23-country validation study [32].

**Patient Health Questionnaire-9 (PHQ-9):** the PHQ-9 [33] is a widely used and validated instrument as diagnostic indicator of depression and a continuous severity score. In Ugandan PCP, the PHQ-9 has a very high sensitivity and specificity, and can be used for screening for depression [34]. A cut-off of ≥10 has been shown to be the optimum cut-off for major depression [33]. The Luganda version of the questionnaire was interviewer-administered.

**Alcohol Use Disorders Identification Test (AUDIT):** to assess the presence of alcohol use disorders in this study, we used the AUDIT [35], which was developed by the World Health Organization (WHO) as a simple method of screening for alcohol use disorders. The AUDIT comprises three domains: hazardous alcohol use (frequency of drinking, typical quantity, and frequency of heavy drinking), dependence symptoms (impaired control over drinking, increased salience of drinking, and morning drinking), and harmful alcohol use (guilt after drinking, blackouts, alcohol-related injuries, and other concerns about drinking). The AUDIT was adapted for local use through the use of pictures and local terms for standard alcohol units. In accordance with previous research in Uganda, a score of 8 or more was considered to be a positive screening result [36]. The Luganda version of the questionnaire was interviewer-administered.

**Multidimensional Scale for Perceived Social Support (MSPSS):** perceived social support was assessed using the MSPSS [37], previously validated for use in Uganda [38]. It contains 12 items, rated on a 7-point Likert-type scale, ranging from 1 “very strongly disagree” to 7 “very strongly agree”. We used the subscale scores for family, friends and/or significant others. Each subscale score ranges from 4 to 28 with a higher score indicating more perceived support. The Luganda version of the questionnaire was interviewer-administered.

**Study procedure:** patients who were willing to participate were interviewed by a clinical officer in a separate room when they were waiting for their consultation.

**Statistical analyses:** continuous data were assessed for normality using the Shapiro-Wilk test and except for the WHODAS 2 and MSPSS scores found not to be normally distributed. For consistency reasons, all correlations with the WHODAS 2 were assessed with a Spearman Rho, while differences in WHODAS 2 total scores between two groups (men versus women, those with versus without a somatic co-morbidity, those with versus without multimorbidity, those with a BMI lower than 18.5 versus those with a BMI equal to or higher than 18.5) were assessed with Student t tests. We corrected for multiple testing using the Bonferroni method in the univariate analyses [39]. The significance level was set at 0.0036 (0.05/14 comparisons). A backward stepwise multivariable regression analysis was performed to evaluate independent variables explaining the variance in WHODAS 2 total scores. To prevent overfitting of the models, only variables significant (P<0.0036) in univariate analyses were entered into the final model. To test for multicollinearity, a variance inflation factor was computed for each independent variable in the model. Values above 3 were used to indicate a multicollinearity problem in the model. A priori, a two-sided level of significance was set at P<0.05 for the regression analysis. Statistical analysis was performed using the statistical package SPSS version 27.0 (SPSS Inc., Chicago, IL).

**Ethical considerations:** the study was conducted in accordance with the Declaration of Helsinki [40]. No compensation was given for participation. Patients were informed that non-participation would not affect their relationship with the primary care center. All participants gave their written informed consent. Participants who could not read, discussed the consent form first with the clinical officer and a relative before providing consent with a fingerprint. The study was approved by the ethical committee of Mengo Hospital and by the Uganda National Council for Science and Technology.

## Results

**Socio-demographic characteristics of participants:** in total, 130 of 139 PCP [median (interquartile range) age=47.0 (22.0); 73.1% (n=95) female] who attended the primary care center during a two-weeks agreed to participate. All 9 potential participants who declined were not interested. There were no missing data. Forty-five PCP did currently not have a job (34.6%). Almost all PCP having a job were farmers (n=82, 96.5%). Two PCP worked as a tailor and one as a cobbler. Seven PCP smoked (5.3%). The median number of cigarettes smoked was 2.0 (interquartile range = 1.0). Fifty-six PCP (43%) reported a chronic somatic condition and /or had a condition registered in the medical files. Reported somatic chronic conditions were low back pain (n=15), ulcerative colitis (n=15), hypertension (n=9), musculoskeletal pain (n=9), cardiovascular disease (n=6), asthma (n=4), migraine (n=3), epilepsy (n=2), urinary tract disease (n=1), diabetes (n=1), thyroid disease (n=1), rheumatoid arthritis (n=1), and uterine problems (n=1). Multimorbidity was present in 13 (10.0%) PCP. Malnutrition defined as a BMI lower than 18.5 was present in 27 PCP (20.8%). The mean (standard deviation) of the WHODAS 2 total score was 13.3 (9.6). Median (interquartile range) of the SIMPAQ, PHQ-9, AUDIT and MSPSS scores are presented in Table 1 [39].

**Table 1:** correlates with the WHODAS 2 in 130 Ugandan primary care patients

Variable	Median (interquartile range)	r with WHODAS-2	P
Age (years)	47.0(22.0)	0.44	<0.001*
Body mass index (kg/m<sup>2</sup>)	21.6(7.0)	-0.28	0.003*
SIMPAQ walking (min/day)	10.0 (25.5)	-0.60	<0.001*
SIMPAQ exercise (min/day)	10.7 (60.0)	-0.63	<0.001*
SIMPAQ incidental (min/day)	60.0 (30.0)	-0.47	<0.001*
PHQ-9 total score	6.0 (3.0)	0.55	<0.001*
AUDIT total score	0.0(1.0)	0.04	0.68
MSPSS family	14.0 (13.0)	-0.30	<0.001*
MSPSS friends	15.0 (10.0)	0.15	0.11
MSPSS significant others	17.0 (12.0)	-0.09	0.30

Due to the limited number of participants smoking (n=7), this variable was excluded from the analyses. *significant when P<0.0036 [39]. AUDIT = Alcohol Use Disorders Identification Test, MSPSS = Multidimensional Scale for Perceived Social Support, PHQ-9 = Patient Health Questionnaire-9, r= Spearman Rho, SIMPAQ = Simple Physical Activity Questionnaire, WHODAS 2 = World Health Organization Disability Assessment Schedule 2.

### Correlates with the WHODAS 2 scores.

**Univariate analyses:** as can be noticed in Table 1 [39], older age, lower BMI, less time spent walking, exercising and in incidental non-structured physical activity, higher levels of depression and lower levels of support from family members were significantly (P<0.0036) associated with more self-perceived disability. No significant associations of harmful drinking and perceived social support from friends or significant non-relatives with self-perceived disability were found. There was no significant difference in WDODAS 2 scores between men and women [respectively 11.6 (8.9) versus 14.0 (10.0), P=0.60], those having versus not having a chronic somatic co-morbidity [respectively 14.8 (9.4) versus 11.9 (9.1), P=0.08], those with versus without multimorbidity [respectively 15.2 (8.1) versus 13.1 (9.8), P=0.47], and those with a BMI lower than 18.5 versus those with a BMI of 18.5 or higher [respectively 16.3 (9.4) versus 12.6 (9.6), P=0.07].

**Multivariate analyses:** significant correlates of the WDODAS 2 in the univariate analyses were included in the backward stepwise regression analysis, i.e., age, BMI, the PHQ-9, MSPSS family and the SIMPAQ scores. No variable had a variance inflation factor of more than 3 and therefore none were removed. Age, SIMPAQ walking and PHQ-9 were independent significant predictors of the variance in WDODAS 2 (Table 2). The final model explained 44.2% of the variance in WDODAS 2

**Table 2:** backward linear regression analysis with the WHODAS 2 as the dependent variable in Ugandan primary care patients (n=130)

Variables	B	SE	β	t	P*	VIF
(Constant)	4.2	2.6	/	1.6	0.11	/
Age (years)	0.1	0.04	0.3	3.7	<0.001*	1.1
PHQ-9 total score	0.8	0.2	0.3	3.8	<0.001*	1.2
SIMPAQ walking (min/day)	-0.2	0.04	-0.3	-4.4	<0.001*	1.2

Only significant correlates were included in the model (i.e. age, body mass index, MSPSS family, SIMPAQ, walking, exercise and incidental physical activity, and PHQ-9 score), *significant when P<0.05, B=unstandardized coefficient, SE= standard error, β=standardized coefficient, PHQ-9 = Patient Health Questionnaire-9, SIMPAQ = Simple Physical Activity Questionnaire, VIF = variance inflation factor, WHODAS 2 = World Health Organization Disability Assessment Schedule 2

## Discussion

The current study demonstrates that in patients attending a primary care center in rural Uganda, older age, lower BMI, less time spent walking, exercising and in incidental non-structured physical activity, higher levels of depression and lower levels of support from family members were significantly associated with more self-perceived disability. Multivariate analyses, on its´ turn, showed that older age, more symptoms of depression, and less time spent walking were independent significant predictors of more self-perceived disability as measured with the WHODAS 2.

While a previous study in older Ugandan people with and without HIV already demonstrated that older age and more symptoms of depression are significant predictors of more self-perceived disability as measured with the WHODAS 2 [41], the current data are the first to report such findings in patients attending a primary care center. Our data are also the first to show that less time spent physically active, and in particular walking, is associated with more self-perceived disability in patients attending primary care centers. The current data consequently support recent calls for a greater focus on investigating holistic collaborative care models considering physical inactivity and depression in primary care settings in rural communities in low-income countries [42]. Since healthcare workers in sub-Saharan Africa encounter challenges in dealing with the increased needs of care and support for PCP who experience disabilities [43], there is need to examine best practices for PCP in rural and remote areas. A recent pilot study [44] in 41 outpatients with mental health problems attending a primary care setting in a remote fishing community in Uganda demonstrated that 8-weeks of once weekly physical activity counselling using the self-determination theory [45] and motivational interviewing [46] framework facilitating autonomy, belongingness and competence resulted in more time spent walking (Cohen´s d = 1.38) and fewer depressive symptoms (Cohen´s d = 1.47), but also more incidental physical activity (Cohen´s d = 1.69), a better physical health (Cohen´s d = 1.38), and better social quality of life (Cohen´s d = 1.39).

Although in the multivariate analyses, social support from family members was not an independent predictor, our univariate analyses indicated that less social support from family members was significantly associated with more self-perceived disability. Our data are in line with a recent study in people with mental health problems in Uganda attending primary care centres demonstrating that increased experienced social support from family members is associated with reduced self-perceived disability [47]. Consequently, future studies exploring holistic collaborative care models should consider evaluating the added value of including family members as well.

Similarly, although in the multivariate analyses, BMI was not an independent predictor, our univariate analyses indicated that a lower BMI was significantly associated with more self-perceived disability. A reason for the observed association might be that a lower BMI can be considered as a measure-of-proxy for a lower socio-economic status in low- and middle-income countries [48], which on its´ turn is also a risk factor for more self-perceived disability [49]. Besides this, it has been demonstrated before as well that lower BMI is in low-income countries such as Uganda associated with chronic conditions such as chronic obstructive pulmonary disease (COPD) [50] and HIV/AIDS [51]. Although we corrected for the presence of chronic somatic comorbidities, it cannot be excluded that due to self-report of these data and / or due to incomplete medical files, the real prevalence was an underestimation. A lack of statistical power might also clarify why no difference in level of self-perceived disability was found between those having versus not having a chronic somatic co-morbidity and those with versus without multimorbidity.

Finally, the reason why harmful drinking was not associated with more self-perceived disability in the univariate analyses might reflect that levels of harmful drinking were very low and consequently there might have been a lack of statistical power here as well. As in a previous community-based studies in Uganda exploring harmful drinking using the AUDIT, it might be that also in our study there was an unwillingness to disclose alcohol use due to the internalized stigma related to it [36,52].

Limitations: although this is the first study to date to collect data on physical activity and self-perceived disability in PCP in a rural area in a low-income country, our results should be interpreted in the light of several limitations. First, because this was a cross-sectional study, causality cannot be inferred. Therefore, it remains unclear whether the self-perceived disability is caused by less physical activity and/or higher levels of depressive symptoms or vice versa. Second, physical activity levels were measured with a self-report questionnaire, which is known to be less accurate than objective assessments [53]. It is well known that self-reported measures can overestimate physical activity levels [54]. Third, whilst we included all chronic somatic conditions which were reported by the participants, other chronic conditions such as cancer may have been present but not identified in the study due to a lack of access to health screening in these underserved rural communities. Therefore, the prevalence of somatic co-morbidity and multimorbidity is likely to be an underestimation and it is possible that the association between somatic co-morbidity, multimorbidity and self-perceived disability could have differed if participants had received additional physical examination. Since information on chronic somatic conditions was mainly based on self-report and medical files were not always complete, reporting bias may exist. Finally, the present study only included data from two primary care centers in one health district, which may limit generalizability at a national level.

## Conclusion

Our study demonstrates that self-perceived disability in PCP living in low-resourced settings is associated with older age, physical inactivity and depressive symptoms. Future lifestyle studies in primary care settings should consider targeting both physical and mental health outcomes in order to reduce self-perceived disability in PCP, in particular in older patients.

### What is known about this topic

- *Non-communicable diseases are increasingly a public health concern in all African countries;*
- *Evidence for collaborative care models targeting non-communicable diseases in primary care settings is still limited in most African countries*.

### What this study adds

- *Non-communicable diseases are increasingly a public health concern in all African countries;*
- *Evidence for collaborative care models targeting non-communicable diseases in primary care settings is still limited in most African countries*.
